# Deep learning-based image classification of turtles imported into Korea

**DOI:** 10.1038/s41598-023-49022-3

**Published:** 2023-12-07

**Authors:** Jong-Won Baek, Jung-Il Kim, Chang-Bae Kim

**Affiliations:** Department of Biotechnology, Sangmyung University, Seoul, 03016 Korea

**Keywords:** Biodiversity, Conservation biology, Machine learning

## Abstract

Although turtles play a key role in maintaining healthy and balanced environments, these are endangered due to global trade to meet the high demand for food, medicine, and pets in Asia. In addition, imported non-native turtles have been controlled as alien invasive species in various countries, including Korea. Therefore, a rapid and accurate classification of imported turtles is needed to conserve and detect those in native ecosystems. In this study, eight Single Shot MultiBox Detector (SSD) models using different backbone networks were used to classify 36 imported turtles in Korea. The images of these species were collected from Google and were identified using morphological features. Then, these were divided into 70% for training, 15% for validation, and 15% for test sets. In addition, data augmentation was applied to the training set to prevent overfitting. Among the eight models, the Resnet18 model showed the highest mean Average Precision (mAP) at 88.1% and the fastest inference time at 0.024 s. The average correct classification rate of 36 turtles in this model was 82.8%. The results of this study could help in management of the turtle trade, specifically in improving detection of alien invasive species in the wild.

## Introduction

Turtles, order Testudines, are major biodiversity components of the ecosystems they inhabit, and often serve as keystone species beneficial to other animals and plants^1,2^. However, most turtles are endangered due to indiscriminate global trade^2,3^. Turtles have traditionally been used for food, medicine, and pets in East and Southeast Asia^4–6^. Consequently, Asia, including China, Hong Kong, Japan, Taiwan, and Korea, imports and exports large quantities of live turtles^7,8^. The annual trade of live turtles in Asia has exceeded 13,000 metric tons, the highest number expected to be collected in the wild^8^. According to the National Institute of Biological Resources (NIBR), the turtle was the second most commonly imported taxon among endangered species that were imported into Korea between 2009 and 2014^9^. In addition, the demand for turtles in Asia has increased in recent decades^8^. In order to satisfy the rising demand for food, medicines, and pets, the illegal trade of turtles has increased^3^. In particular, due to pet preference, the illegal trade of rare turtle species, such as *Cuora flavomarginata*, increases^10,11^. Various international conventions and conservation bodies, including the Convention on International Trade in Endangered Species of Wild Fauna and Flora (CITES) and the International Union for the Conservation of Nature and Natural Resources (IUCN), seek to protect turtles from extinction and illegal trade.

The wildlife trade has been known to be a source of alien invasive species^12–14^. These negatively affect native species through predation and competition, diffuse pathogens, and modify the functioning of the ecosystem and abiotic features of environments^13,15,16^. In particular, turtles have a greater impact on the native population than other animals as they are long-lived organisms which can survive for decades in suboptimal habitats where environmental characteristics are suitable for breeding^17,18^. The introduction of alien turtles into the wild has continued due to abandonment of breeding as pets, religious release, and careless management^11,19,20^. Alien invasive turtles have been reported in various countries. Since 1997, the European Union has prohibited the import of slider turtle, *Trachemys scripta elegans*, because it competes for food and basking spots with the threatened European pond turtle, *Emys orbicularis*^21,22^. Native to the Americas, *Chelydra* and *Macrochelys*, are found in the Yangtze River basin in China^23^. The snapping turtle, *Chelydra serpentina*, was imported to Japan from the American continent as a pet, but some have been and continue to be abandoned in the wild^24^. In Korea, invasive turtles have also been reported, where non-native turtles such as *Graptemys ouachitensis* and *Macrochelys temminckii* have been observed^25,26^. These are now included on the NIBR’s list of imported turtle species^9^. In order to preserve native ecosystems, it is essential to detect and classify these non-native turtles coming no through the pet trade.

To identify which species are being traded, species identification based on morphological features has been used as a standard and effective method^27–29^. However, this requires skilled experts to identify targeted wildlife^28^, whose numbers have declined in recent years^30^. In this regard, the identification of turtles, particularly the genera *Chelonoidis* and *Geochelone*, is not an easy task due to similar morphological features between species^31,32^. Additionally, the skeletal traits used to identify turtles can be altered by ecological factors, including food and behavior^33,34^. These challenges make the identification of turtles based on their morphological features difficult.

Early Detection and Rapid Response (EDRR) are recognized as a set of actions that increase the chances of invasive species containment and eradication before they enter irreversible stages of the invasion curve^35^. Currently, the detection of invasive species through the camera trap method has been widely conducted for EDRR^36^. However, this method has become more challenging due to the manual analysis used to identify species based on morphology using a large image dataset^37^. Therefore, rapid and accurate tools are needed to identify species among the large quantities imported via the turtle trade.

Molecular methods, such as DNA barcoding, have been suggested as tools to control the wildlife trade and manage invasive species^38–41^. Current approaches to DNA barcoding necessitate laboratory analysis and require significant time and expertise to examine samples^36,42^. Therefore, a rapid and accurate method is required to detect imported turtles based on morphological features.

Image classification based on deep learning is expected to improve the ability to identify traded wildlife and detect alien invasive species^43,44^. This method can reduce personnel and time spent identifying species in the wildlife trade^44^. Moreover, this can be used to verify the precision of species occurrence data, leading to improved cost-effectiveness in data administration and precise insights for decision support tools designed to detect alien invasive species^36,37^. A Convolutional Neural Network (CNN) is a deep learning model developed for the classification of image data. Object detection models are developed based on CNNs and consider classification and regression, allowing for the prediction of objects in images. These models have been widely applied to the automatic classification of species of various organisms^45–47^. The object detection model can be divided into two and one-stage detectors. A two-stage detector, such as Faster-R-CNN^48^, learns regression and classification independently and continuously, whereas a one-stage detector, such as Single Shot MultiBox Detector (SSD)^49^, learns regression and classification simultaneously. These characteristics imply that one-stage detectors are better suited for real-time species classification of large quantities of wildlife trade^47^.

In the present study, object detection models, SSD using eight different CNNs as backbone networks, were assessed to classify turtles imported into Korea. The performance of the eight models was evaluated by the mean Average Precision (mAP) and inference time it takes to process one image. The model test results, which showed the highest mAP, are presented as a confusion matrix.

## Results

### Performance of the eight models

**Figure 1:**
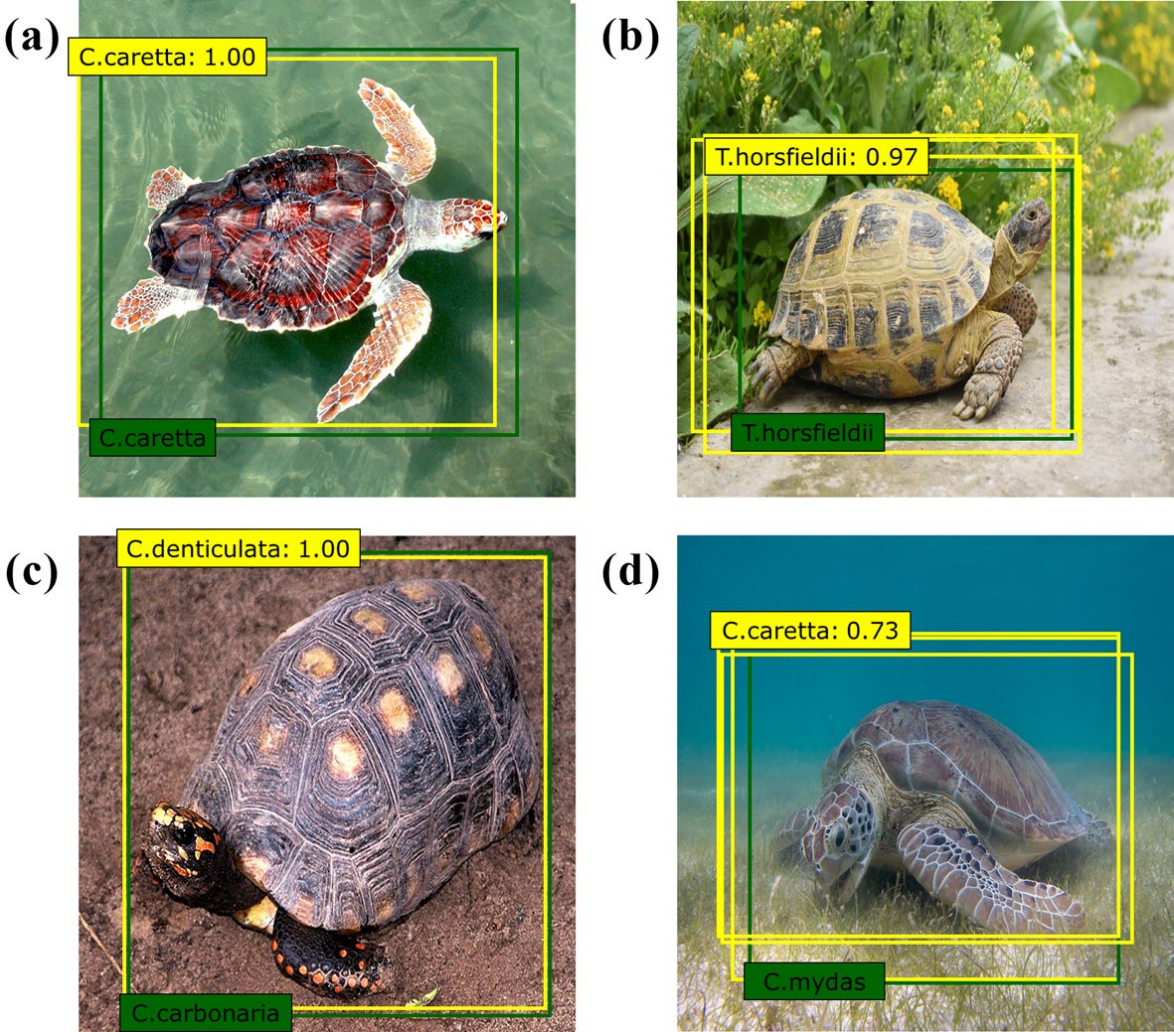
Representative images of four prediction result cases by the models. The yellow and green boxes on the images represent the prediction boxes and the ground-truth bounding boxes, respectively. The values in the yellow boxes of the images are confidence scores suggested by the models, indicating the probability that the prediction is correct. (**a**) Image of *Caretta caretta,* where one prediction bounding box was predicted and classified correctly, (**b**) Image of *Testudo horsfieldii,* where multiple prediction bounding boxes were predicted, and the classification result with the highest confidence score provided by the model was correct, (**c**) Image of *Chelonoidis carbonaria,* where one prediction bounding box was predicted and classified incorrectly, (d) Image of *Chelonia mydas*, where multiple prediction bounding boxes were predicted, and the classification result was incorrect. Photo credit: (**b**) Amirekul, (**c**) Dick Culbert, (**d**) P.Lindgren.

**Figure 2:**
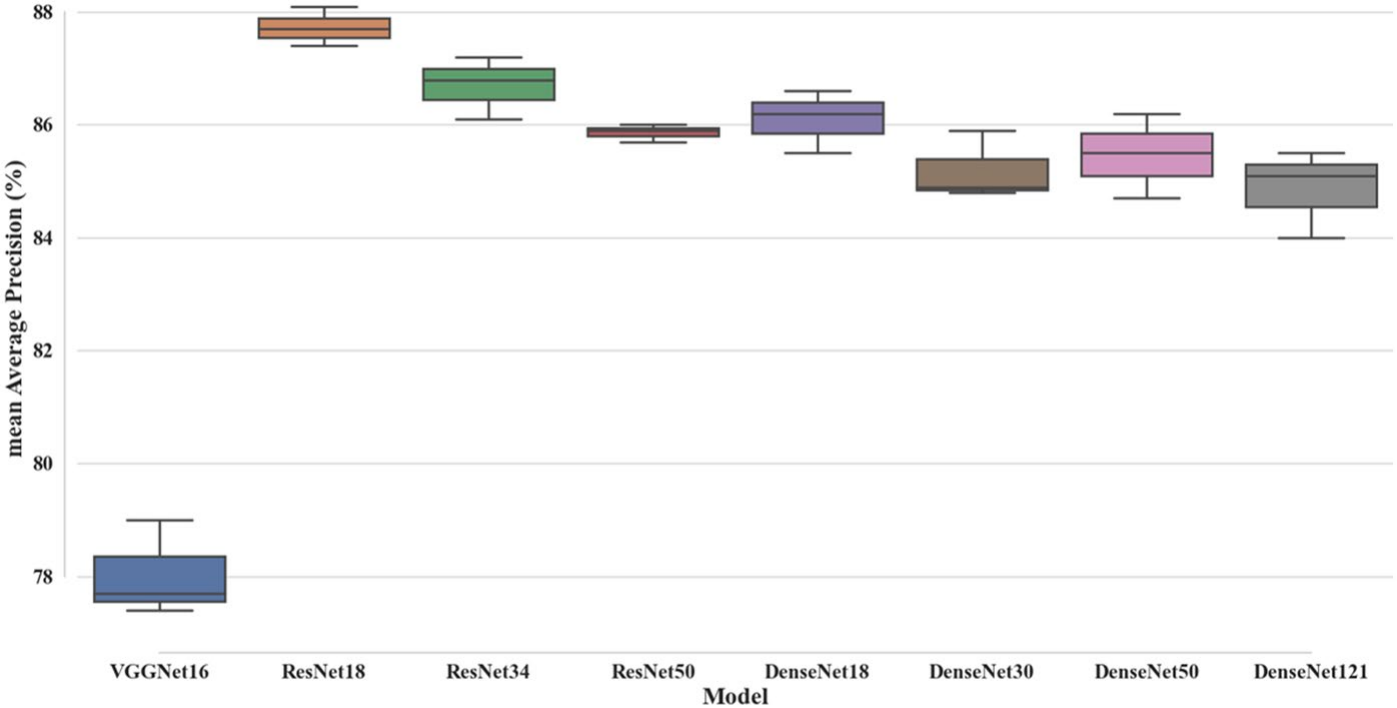
Comparison of the mean Average Precision (mAP) of the eight SSD models for classifying 36 turtles.

Figure 1 depicts representative prediction results from the eight models. There were four cases of prediction results of the eight models. First, one prediction bounding box was predicted and classified correctly (Fig. 1a). Second, multiple prediction bounding boxes were predicted, and the classification result with the highest confidence score provided by the model was determined to be correct (Fig. 1b). Third, a prediction bounding box was predicted and misclassified (Fig. 1c). Fourth, multiple prediction bounding boxes were expected, and it was determined that the classification result with the highest confidence score provided by the model was incorrect (Fig. 1d). Performances of the eight SSD models included in different CNN backbone networks are shown in Fig. 2 and Table S1. The mAP of the models varied based on the CNN used as the backbone network (Fig. 2). Among the three ResNet models, the ResNet18 model had the highest mAP, while the ResNet50 model had the lowest mAP (Fig. 2). The DenseNet30 model had the lowest mAP among the four DenseNet models, while the DenseNet18 model had the highest mAP (Fig. 2). Among all training results, the highest mAP was 88. 1% of the ResNet18 model, and the lowest was 77.4% of the VGGNet16 model (Table S1). In the VGGNet16 model, which showed the lowest mAP, *Chelodina mccordi* presented the lowest AP at 55.0%, and *Clemmys guttata* presented the highest AP at 97.0% (Table S2). *Chelonia mydas* had the lowest AP (69.8%), while that of *Stigmochelys pardalis* was the highest (97.6%) in the ResNet18 model, which showed the highest mAP (Table S2). The inference time ranged from 0.024 s for the ResNet18 model to 0.052 s for the DenseNet121 model (Table S1).

### Classification results of the model which showed the highest mAP

The confusion matrix displays the classification results of the ResNet18 model, which had the highest mAP (Table 1). This model correctly classified 36 turtles with an average accuracy of 82.8%, ranging from 60.7% for *Chelonia mydas* to 100.0% for *Stigmochelys pardalis* (Table 1). Nine cases showed a more than 10.0% misclassification rate (Table 1). At 26.0%, the classification of *Geochelone elagans* as *Geochelone platynota* was the model’s most incorrect classification. Similarly, 25.0% of test images of *Geochelone platynota* were incorrectly classified as *Geochelone elagans*. The misclassification rate of *Chelonoidis chilensis* as *Geochelone sulcate* was 16.7%. The incorrect classification of *Chelonia mydas* as *Caretta caretta* and *Eretmochelys imbricata* was 14.3%, respectively. *Chelonoidis denticulata* and *Caretta caretta* were misclassified as *Chelonoidis carbonaria* and *Chelonia mydas* at 12.5%, respectively. 10.7% of *Cuora amboinensis* were misclassified as *Mauremys sinensis*. Additionally, *Chelonia mydas* had the lowest correct classification rate due to its misclassification as *Caretta caretta* and *Eretmochelys imbricata*.

**Table 1:**
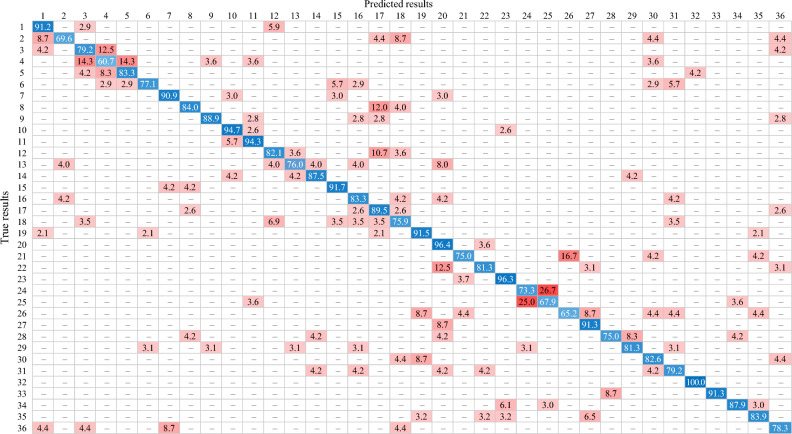
The confusion matrix of the ResNet18 model, showing the highest mean average precision (mAP).

The numbers 1 to 36 indicate the 36 turtles, as shown in Table 1. The rows contain the actual species, while the columns contain the species predicted by the models. The prediction results for the models are shown as percentage values, while the zero values are presented as a Endash. The correct predictions are shaded in blue, and the incorrect predictions are shaded in red; the deeper the blue, the higher the value of the correct prediction, and the deeper the red, the higher the value of the incorrect prediction.

## Discussion

The models using ResNet and DenseNet as backbone networks exhibited higher mAP than the using VGGNet models (Fig. 2). The difference may be attributable to the more complex architectures of the ResNet and DenseNet models compared to that of the VGGNet model. ResNet models enhanced network performance by resolving the degradation issue inherent to the VGGNet model used in previous layers by utilizing a skip connection that can jump over layers and add features^50^. DenseNet models connect all layers directly and reuse all previous layers’ features to maximize information delivery^51^. In addition, a direct correlation was discovered between inference time and model complexity, with the exception of the VGGNet16 model (Table S1). The more layers a model had, the longer it took to make inferences. The DenseNet models showed a slower inference time than those using ResNet as backbone networks.

**Figure 3:**
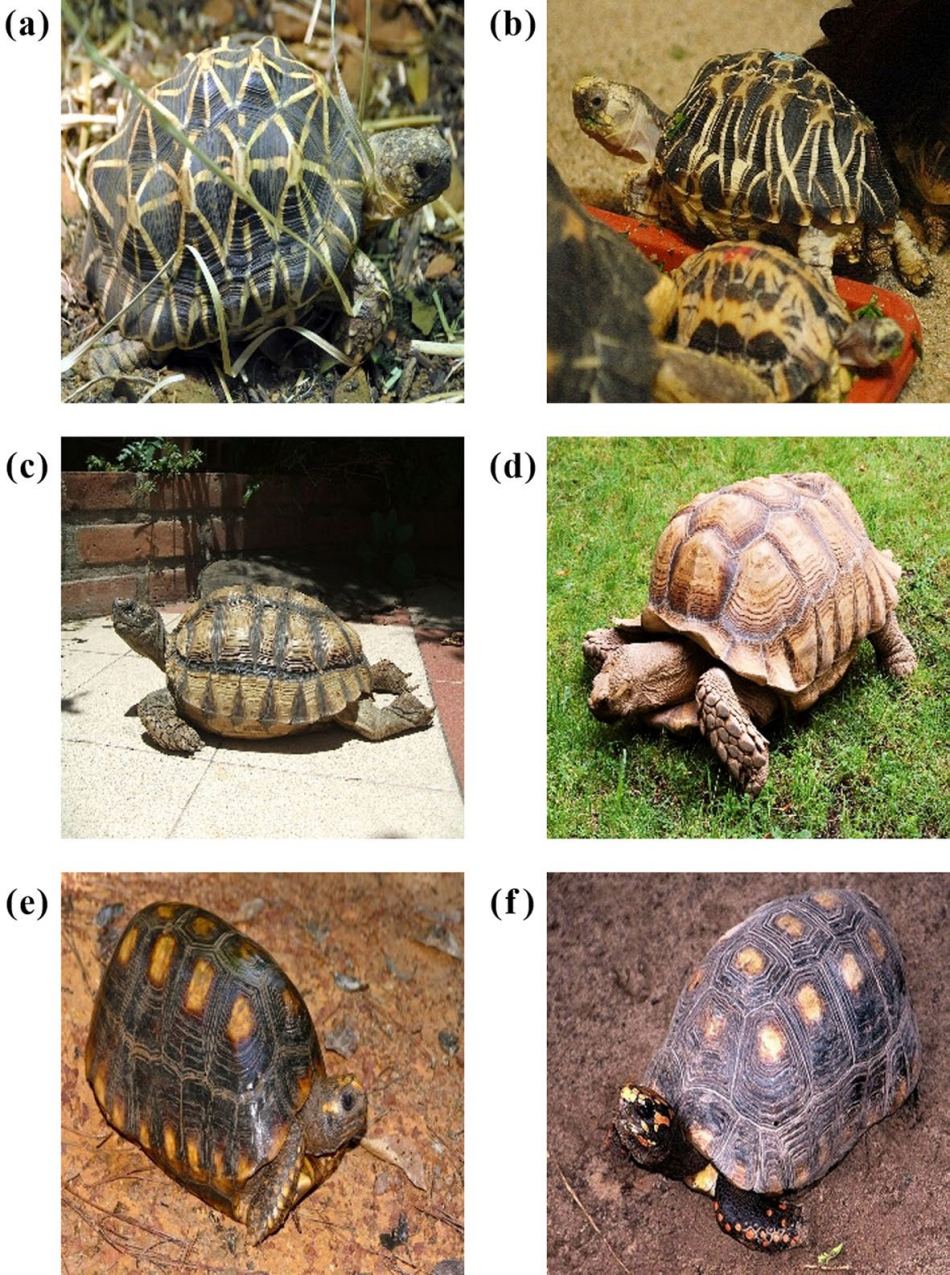
Representative images of the incorrect classification results of the ResNet18 model caused by morphological similarities between species. (**a**) *Geochelone elegans*, (**b**) *Geochelone platynotan*, (**c**) *Chelonoidis chilensis*, (**d**) *Geochelone sulcata*, (**e**) *Chelonoidis denticulate*, (**f**) *Chelonoidis carbonaria*. Photo credit: (**a**) Tatarinov A. C., (**b**) Jacob.jose, (**c**) Arteivanna, (**d**) MartinThoma, (**e**) Bernard DUPONT, (**f**) Dick Culbert.

The ResNet18 model showed the highest mAP at 88.1% among the eight models examined in this study (Fig. 2), while nine of the misclassification results from the ResNet18 model showed a rate of more than 10.0% (Table 2). Four of these nine misclassification cases could be due to the morphological similarity between species. Representative images of these misclassification cases are presented in Fig. 3. Morphological identification between *Geochelone elegans* and *Geochelone platynota*, which had the highest misclassification rate by the model, has been known to be challenging (Figs. 3a and b)^32^. These species have star-like patterns on their shells, and their carapace is dark brown with light yellow radiating markings^32,52^. However, the color of the top of the head makes *Geochelone elegans* and *Geochelone platynotan* distinguishable. Although *Geochelone elegans* has a yellow and black top of the head with small scales, *Geochelone platynota* has a primarily yellow top of the head with large scales^32,52^. In order to increase the classification accuracy between these species, images that showed morphological differences between the two species should be added to train the models. Additionally, although *Chelonoidis chilensis* and *Geochelone sulcata* belong to different genera, the misclassification of these could be due to morphological similarity (Fig. 3c and d). Furthermore, these species are similar due to their black annuli with brown–yellow lines on the carapace^52,53^. The convex scutes on the carapace and the serrated anterior and posterior margins of the shell of *Geochelone sulcata* make this species distinguishable from *Chelonoidis chilensis*^52,53^. Therefore, the collection of images of *Geochelone sulcata* that showed the shape of scutes and shell’s margins should increase this species’ correct classification rate. *Chelonoidis carbonaria* is difficult to classify because its morphological characteristics are very similar to those of *Chelonoidis denticulata* (Fig. 3e and f)^31^. Both species lack a cervical scute on their carapace. Their carapaces are black with yellow or orange patches. However, these species can be distinguished based on head characteristics^52,53^. In contrast, while the head of *Chelonoidis carbonaria* is yellow to red with one large frontal scale, the head of *Chelonoidis denticulata* has multiple frontal scales and large yellow or orange scales at the top of the head^52,53^.

**Table 2:** The dataset of turtle species examined in this study.

Number	Family	Species	Training set	Validation set	Test set
1	Carettochelyidae	*Carettochelys insculpta*	156	33	34
2	Chelidae	*Chelodina mccordi*	105	22	23
3	Cheloiniidae	*Caretta caretta*	109	23	24
4	Cheloiniidae	*Chelonia mydas*	126	27	28
5	Cheloiniidae	*Eretmochelys imbricata*	106	22	24
6	Cheloiniidae	*Macrochelys temminckii*	158	33	35
7	Emydidae	*Clemmys guttata*	150	32	33
8	Emydidae	*Graptemys ouachitensis*	109	23	25
9	Emydidae	*Malaclemys terrapin*	164	35	36
10	Emydidae	*Terrapene carolina*	173	37	38
11	Emydidae	*Terrapene ornata*	157	33	35
12	Geoemydidae	*Cuora amboinensis*	124	26	28
13	Geoemydidae	*Cuora flavomarginata*	110	23	25
14	Geoemydidae	*Cuora galbinifrons*	108	23	24
15	Geoemydidae	*Geoclemys hamiltonii*	106	22	24
16	Geoemydidae	*Heosemys spinosa*	107	23	24
17	Geoemydidae	*Mauremys sinensis*	173	37	38
18	Podocnemididae	*Podocnemis unifilis*	128	27	29
19	Testudinidae	*Aldabrachelys gigantea*	214	45	47
20	Testudinidae	*Chelonoidis carbonaria*	252	54	55
21	Testudinidae	*Chelonoidis chilensis*	112	24	24
22	Testudinidae	*Chelonoidis denticulata*	143	30	32
23	Testudinidae	*Chersina angulata*	121	26	27
24	Testudinidae	*Geochelone elegans*	135	29	30
25	Testudinidae	*Geochelone platynota*	125	26	28
26	Testudinidae	*Geochelone sulcata*	105	22	23
27	Testudinidae	*Gopherus berlandieri*	105	22	23
28	Testudinidae	*Indotestudo elongata*	107	22	24
29	Testudinidae	*Malacochersus tornieri*	147	31	32
30	Testudinidae	*Manouria emys*	105	22	23
31	Testudinidae	*Manouria impressa*	109	23	24
32	Testudinidae	*Stigmochelys pardalis*	192	41	42
33	Testudinidae	*Testudo horsfieldii*	166	22	23
34	Testudinidae	*Testudo hermanni*	86	31	33
35	Testudinidae	*Testudo marginata*	142	30	31
36	Trionychidae	*Lissemys punctata*	105	22	23
Total			4840	1023	1071

The classifications between three sea turtles, *Chelonia mydas*, *Caretta caretta*, and *Eretmochelys imbricata*, were not clear, despite the distinguishable morphological features of these species (Table 1). A pair of prefrontal scales and one claw per limb make *Chelonia mydas* distinguishable from the other two sea turtles^52,53^. The misclassifications among these species could be due to the underwater images used to train the models. The use of underwater images presents challenges such as color bias, unclear image quality, and image quality degradation^54^. Methods for enhancing underwater image have been developed, including the histogram-based method, which can improve the visual appearance by manipulating pixels directly without regard to the imaging principle^55,56^. In future studies, methods for enhancing underwater image quality should be applied. In order to improve the classification accuracy of these sea turtles, images depicting their morphological characteristics should be added to the training data for the classification models. The misclassification of *Graptemys ouachitensis* as *Mauremys sinensis* and *Cuora amboinensis* as *Mauremys sinensis* could be due to the lack of images to train the models. *Graptemys ouachitensis* and *Mauremys sinensis* can be easily distinguished by small yellow spots on the upper and lower jaws of *Graptemys ouachitensis*^52^. *Cuora amboinensis* can be distinguished from *Mauremys sinensis* by three bright yellow stripes on the sides of the head of *Cuora amboinensis*^52,53^. More images should also be included to train the models to increase the classification accuracy of *Graptemys ouachitensis* and *Cuora amboinensis*.

Despite the importance of classifying globally traded turtles to conserve and detect invasive turtle species, there are few studies on automatic and accurate classification of those^57–59^. These previous studies applied deep learning models to detect or classify two to five species of turtles. Furthermore, most studies have been conducted to detect sea turtles rather than classify those^58,59^. Unlike previous studies, deep learning models were evaluated to classify 36 turtles, including sea turtles, freshwater turtles, and tortoises, imported into Korea in the present study. Moreover, this is the first study to classify turtles, including endangered species, imported into Asian countries. Among the turtles examined in this study, eight species are categorized as Critically Endangered, nine are Endangered, and 11 are Vulnerable, according to the IUCN Red List (https://www.iucnredlist.org) (Table S3). Additionally, according to the CITES Checklist (https://www.checklist.cites.org), eight species are listed in Appendix I, where their trade is prohibited, and 26 are in Appendix II, where their trade is closely monitored through permits (Table S3). Furthermore, two species, *Macrochelys temminckii,* and *Graptemys ouachitensis*, examined in this study are reported as alien invasive turtles in Korea^25,26^. Therefore, the novelty of this study is that the models can be used to control the global trade of endangered turtles for conservation purposes and to detect alien invasive turtles in the native ecosystem for EDRR.

Although this study applied the object detection model to classify turtles, it has limitations. Typically, object detection models focus on detecting and classifying multiple objects in images. However, this study’s dataset primarily consists of a single object in the images. Therefore, it is necessary to collect images of multiple objects at various scales, and test models using these images in further research. Given that the images for this study were obtained from the Internet also presents a challenge, which made it difficult to collect a comprehensive set of images of turtles in various poses, which could reduce the classification accuracy.

In further studies, a comprehensive collection of turtle images should be conducted using various methods, such as taking a photo directly at customs. Furthermore, future studies should include more turtles that are frequently traded worldwide. Object detection models are still being developed, for example, EfficientDet^60^ and YOLOv5^61^. Thus, it is necessary to apply the other models to find a more suitable model to classify turtles. Recently, transfer learning has been applied to increase the classification accuracy of various organisms^62,63^. This can transfer the knowledge from the pre-trained model using vast data, such as the ImageNet benchmark dataset, to the new task model^64^. The accuracy of the model developed in this study will be increased using transfer learning in future studies. Finally, the model will be supplied as a mobile application for use in customs control of the turtle trade and in the wild for the detection of invasive turtles.

## Conclusion

In this study, the eight SSD models with different CNN backbone networks were evaluated to classify 36 turtle species. Among the eight models, the ResNet18 model showed the highest mAP and the fastest inference time. This model classified the 36 turtles correctly with an average accuracy of 82.8%, ranging from 60.7% for *Chelonia mydas* to 100.0% for *Stigmochelys pardalis*. The models developed in this study have the potential to automatically and accurately classify turtle species among the vast quantities of traded turtles. Application of this model can aid in regulating turtle trade for conservation purposes and detecting the alien invasive turtles in native ecosystems for EDRR. In order to improve the classification accuracy of the models in future studies, a comprehensive collection of turtle images should be considered despite the novelty of this study. Databases providing images of various species taken by citizen scientists, such as iNaturalist (https://www.inaturalist.org/), might be helpful in comprehensive image collection. Future research should also employ other recently developed object detection models to identify a suitable classification scheme for turtles. Furthermore, transfer learning will be used to increase the accuracy of classification. Finally, the model will be supplied as a mobile application.

## Methods

**Figure 4:**
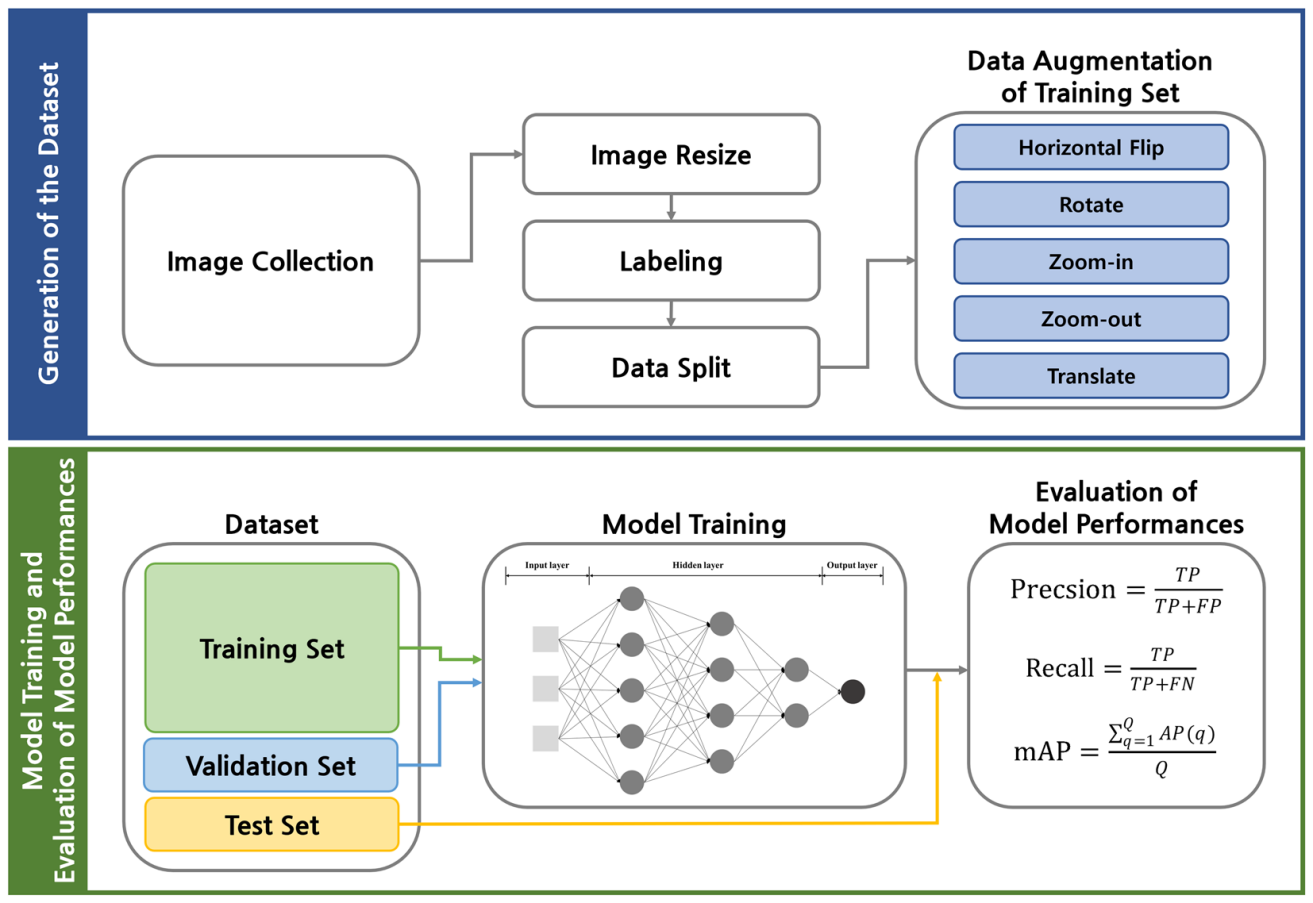
The flowchart of the overall methodology for classifying 36 turtles. (1) Generation of turtle image dataset, and applying data augmentation on the training set, (2) Model training using the training and validation sets, and evaluation of trained model performances using the test set.

The entire methodology of this study is presented as a flowchart in Fig. 4. Images of sea turtles imported into Korea were collected. These were integrated into the size required by the SSD model used in the study, and the object in the images was labeled. The images were split into the training, validation, and test sets, and data augmentation was applied to the training set. The models were trained using the final dataset established in this study, and the performances of the models were evaluated.

### Collection of image data

**Figure 5:**
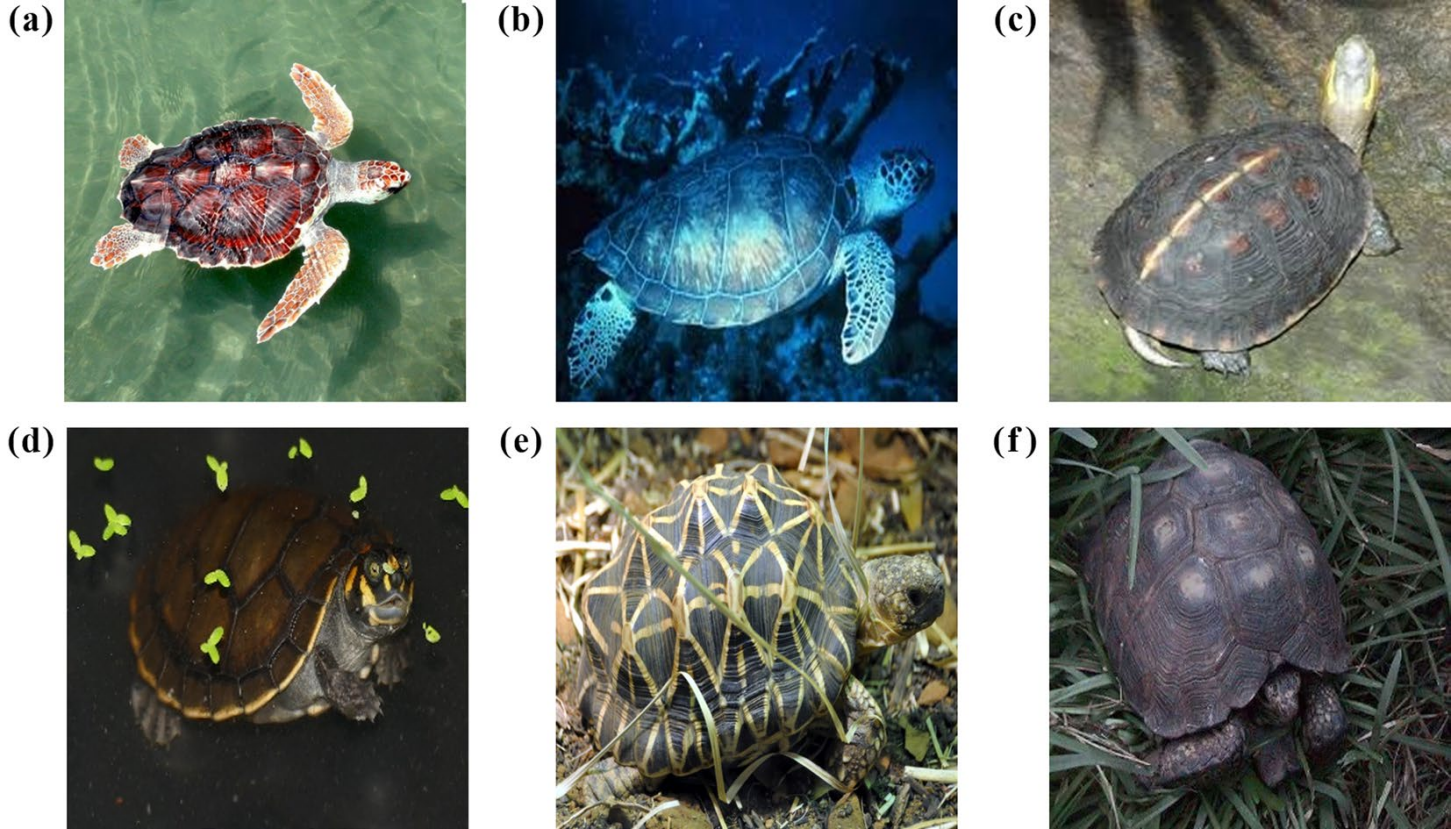
Representative images of examined species with various backgrounds. (**a**) *Caretta caretta*, (**b**) *Chelonia mydas*, (**c**) *Cuora flavomarginata*, (**d**) *Podocnemis unifilis*, (**e**) *Geochelone elegans*, (**f**) *Gopherus berlandieri*. Photo credit: (**d**) Bernad DUPONT, (**e**) Jacob.jose, (**f**) Dawson.

Images of 51 turtle species that are imported into Korea were collected from the Internet because there was no standard dataset for these species. Scientific and common names were used as keywords for comprehensive image collection. The image collection from the Internet has been used to establish a dataset for deep learning to obtain images of various individuals with diverse backgrounds in the absence of a standard dataset^47,65–67^. Turtle images were collected at the species level due to a lack of data at the subspecies level. The images were identified using morphological features described in scholarly books, such as the color and shape of carapaces, the absence or presence of scute, the scale’s relative size, and the number of claws per limb^32,52,53^. While sex dimorphism in turtles is mainly due to differences in size^68^, *Graptemys ouachitensis* and *Podocnemis unifilis* have morphological differences depending on sex^69,70^. Therefore, images of males and females of these two species were included in the dataset. Images in which species could not be identified or had low quality were excluded from the dataset. This study included all species with more than 150 initial images. In the end, 36 species from 8 families and 26 genera were chosen for the study after meeting the outlined criteria (Table 2). Table S3 displays the native distribution, the IUCN Red List category, and the CITES Appendix of the turtles examined in the present study. Except for *Caretta caretta*, *Chelonia mydas*, and *Eretmochelys imbricata*, none of the examined species are indigenous to Korea (Table S3). Figure 5 depicts representative images of examined species with various backgrounds and angles. Marine (Fig. 5a and b), freshwater (Fig. 5c and d), and grassland (Fig. 5e and f) images depict the habitat characteristics of turtles. In addition, the images from various angles, such as dorsal (Fig. 5a), dorsal lateral (Fig. 5b and e), dorsal posterior (Fig. 5c), and dorsal frontal (Fig. 5d and f), are included. The images were integrated into a 300 × 300-pixel image which was the size required by the object detection model used in the study. Additionally, the horizontal and vertical resolutions of the images were converted to 96 DPI. The ground-truth bounding boxes have designated the entire body using DarkLabel (https://darkpgmr.tistory.com/16) since the morphological features that classify the turtles are located all over the bodies. The dataset was divided into a 70% training set, a 15% validation set, and a 15% test set. Subsequently, data augmentation was applied to the training set, which included horizontal flip, random horizontal and vertical translation between − 30 and 30 pixels, random rotation between − 10° and 10°, and random zoom-in and zoom-out between 100 and 200%. At least 10,000 images of the examined species were included in the training set through data augmentation (Table S4).

### Model training

**Figure 6:**
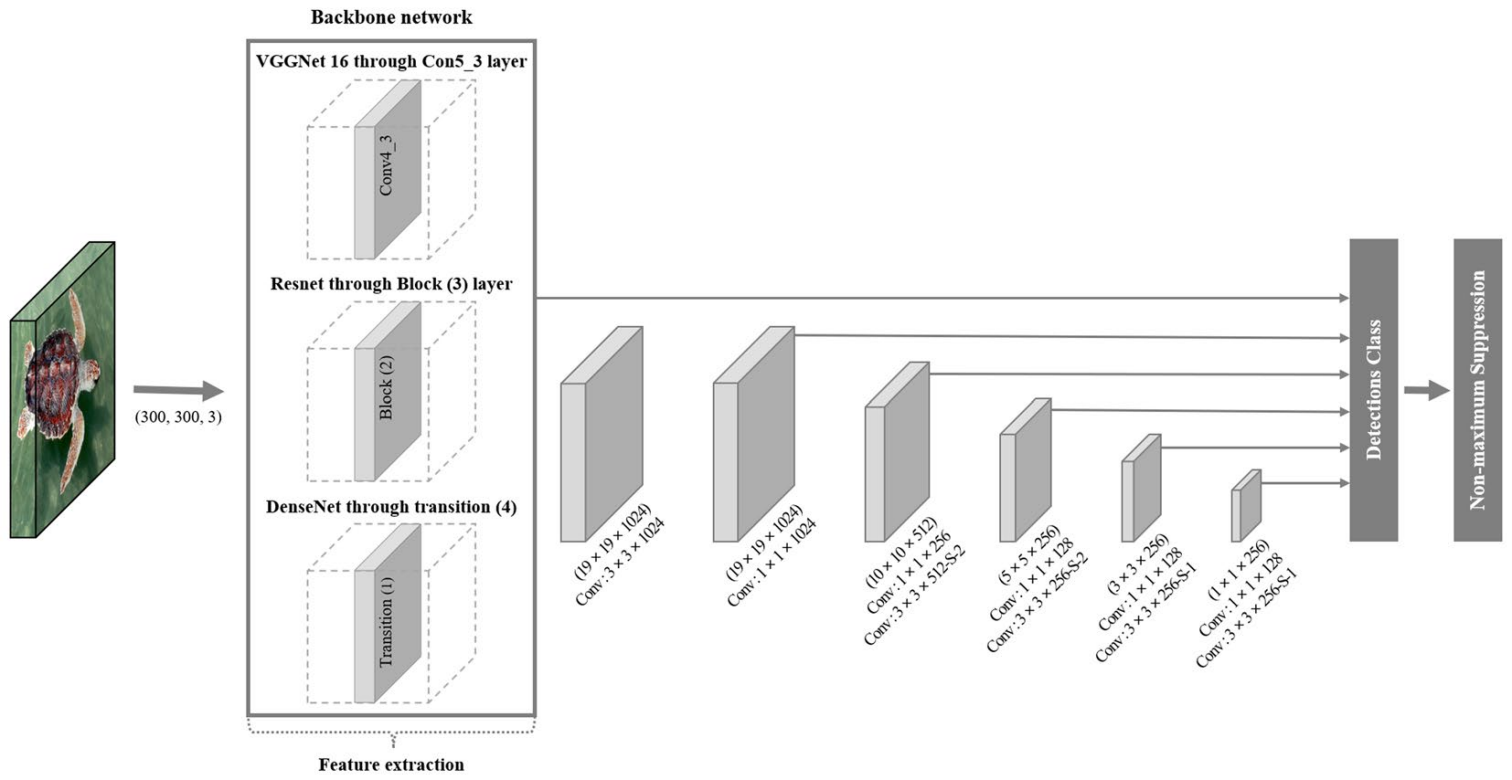
Architecture of the Single Shot MultiBox Detector (SSD) model with different Convolutional Neural Networks (CNN) as the backbone network to classify the 36 turtles.

In this study, the SSD model was used to classify 36 turtle species (Fig. 6). Regression and classification were applied using a convolution layer on a multi-scale feature map. As backbone networks, eight CNN models were used in total. There is one VGGNet model^71^, three ResNet models^50^, and four DenseNet models^51^ among the eight CNN backbone networks. These models’ structures are presented in Tables S5–S7. The same environment and equipment were used to train eight SSD models. These models’ experimental platform is based on the Ubuntu 20.04 operating system and includes RTX 2080 Ti Graphics with 11G video memory, two Intel Xeon Silver 4110 CPUs, and 16 GB of REG.ECC DDR4 SDRAM. The experimental program is written in Python 3.9.7 and runs on the software PyCharm 2021.1 in Keras-TensorFlow environments. The early stop function of Keras was employed to prevent overfitting. In order to ensure consistency, each of the eight models was trained three times.

### Evaluation of model performances for detection and classification of object

The performance of the model was assessed using mean Average Precision (mAP) and inference time. Average Precision (AP) values for each class were determined using precision-recall curves obtained with precision (true positive/true positive + false positive) and recall (true positive/true positive + false negative) measurements. The Intersection over Union (IoU) was used to determine the overlap ratio between the hand-labeled ground-truth bounding boxes and the predicted bounding boxes suggested by the model. When a model made a prediction with an IoU threshold value greater than the value determined by the researcher, the prediction was deemed to be a true positive. The IoU threshold value was determined as 0.5 in this study. Finally, the mAP value was calculated using formula (1), where *Q* is the number of queries of the dataset, and AP(*q*) is the AP for the given query *q*.

1 $$\mathrm{mean} \, \mathrm{ Average } \, \mathrm{Precision} \, \left(\mathrm{mAP}\right)= \frac{\sum_{q=1}^{Q}\mathrm{AP}\left(q\right)}{Q}$$

The inference time was calculated as the time taken to process one image. Finally, the classification results of the model showing the highest mAP are represented in a confusion matrix. The classification result with the highest confidence value was chosen when models predicted multiple classification results.

### Supplementary Information

Supplementary Tables.

## Data Availability

The datasets examined in this study are available from the corresponding author upon a reasonable request.
