# Supplementary material for: Deep learning-based image classification of turtles imported into Korea
Source: Sci Rep. 2023 Dec 7;13:21677. doi: 10.1038/s41598-023-49022-3 (PMC10709346; doi:10.1038/s41598-023-49022-3)
Supplement: Supplementary file 1 — Supplementary Tables. [file 41598_2023_49022_MOESM1_ESM.docx]

**Supplementary Materials**

**Deep learning-based image classification of turtles imported into Korea**

Jong-Won Baek†, Jung-Il Kim†, and Chang-Bae Kim^*^

Department of Biotechnology, Sangmyung University, Seoul 03016, Korea

†These authors contributed equally to this work.

*****Email: evodevo@smu.ac.kr

**Table S1.** The mean Average Precision (mAP) and inference time of the eight models examined in this study.

| **Model** | | **Run 1** | **Run 2** | **Run 3** | **Average** | **Standard  Deviation** |
| --- | --- | --- | --- | --- | --- | --- |
| VGGNet16 | mAP (%) | 77.7 | 77.4 | 79.0 | 78.0 | 0.7 |
|  | Inference time (s) | 0.029 | 0.028 | 0.029 | 0.029 | 0.000 |
| ResNet18 | mAP (%) | 87.7 | 87.4 | 88.1 | 87.7 | 0.3 |
|  | Inference time (s) | 0.024 | 0.023 | 0.024 | 0.024 | 0.000 |
| ResNet34 | mAP (%) | 87.2 | 86.8 | 86.1 | 86.7 | 0.5 |
|  | Inference time (s) | 0.027 | 0.029 | 0.030 | 0.029 | 0.001 |
| ResNet50 | mAP (%) | 85.9 | 85.7 | 86.0 | 85.9 | 0.1 |
|  | Inference time (s) | 0.031 | 0.033 | 0.032 | 0.032 | 0.001 |
| DenseNet18 | mAP (%) | 86.6 | 85.5 | 86.2 | 86.1 | 0.5 |
|  | Inference time (s) | 0.033 | 0.033 | 0.032 | 0.033 | 0.000 |
| DenseNet30 | mAP (%) | 84.8 | 84.9 | 85.9 | 85.2 | 0.5 |
|  | Inference time (s) | 0.036 | 0.034 | 0.036 | 0..035 | 0.001 |
| DenseNet50 | mAP (%) | 84.7 | 85.5 | 86.2 | 85.5 | 0.6 |
|  | Inference time (s) | 0.047 | 0.045 | 0.045 | 0.046 | 0.001 |
| DenseNet121 | mAP (%) | 85.5 | 84.0 | 85.1 | 84.9 | 0.6 |
|  | Inference time (s) | 0.052 | 0.051 | 0.050 | 0.051 | 0.001 |

**Table S2.** The average precision (AP) of each species of the eight models, which showed the highest mean average precision (mAP) among the three set of training.

| **Number** | **Species** | **Average Precision (%) of the models** | | | | | | | |
| --- | --- | --- | --- | --- | --- | --- | --- | --- | --- |
|  |  | **VGGNet16** | **ResNet18** | **ResNet30** | **ResNet50** | **DenseNet18** | **DenseNet30** | **DenseNet50** | **DenseNet121** |
| 1 | *Carettochelys insculpta* | 77.0 | 95.3 | 94.9 | 90.7 | 89.5 | 85.9 | 80.2 | 90.7 |
| 2 | *Chelodina mccordi* | 55.0 | 83.3 | 74.8 | 77.3 | 79.5 | 78.8 | 90.2 | 76.9 |
| 3 | *Caretta caretta* | 66.5 | 84.6 | 74.0 | 72.1 | 81.1 | 81.5 | 79.8 | 79.6 |
| 4 | *Chelonia mydas* | 65.9 | 69.8 | 71.4 | 65.8 | 75.8 | 67.1 | 76.4 | 62.8 |
| 5 | *Eretmochelys imbricata* | 72.4 | 88.5 | 84.0 | 74.2 | 80.0 | 81.7 | 81.0 | 75.5 |
| 6 | *Macrochelys temminckii* | 67.0 | 91.2 | 91.7 | 91.5 | 85.1 | 79.0 | 76.5 | 82.7 |
| 7 | *Clemmys guttata* | 97.0 | 95.6 | 96.4 | 94.7 | 96.2 | 96.6 | 96.4 | 91.3 |
| 8 | *Graptemys ouachitensis* | 75.1 | 87.1 | 90.0 | 91.8 | 91.7 | 90.9 | 85.9 | 87.8 |
| 9 | *Malaclemys terrapin* | 80.3 | 94.1 | 88.7 | 91.4 | 94.1 | 93.6 | 83.8 | 84.0 |
| 10 | *Terrapene carolina* | 90.7 | 94.6 | 97.1 | 94.3 | 96.5 | 97.2 | 93.3 | 95.9 |
| 11 | *Terrapene ornata* | 88.4 | 94.7 | 89.5 | 89.9 | 92.9 | 94.5 | 92.0 | 92.4 |
| 12 | *Cuora amboinensis* | 76.1 | 84.1 | 79.8 | 77.7 | 84.9 | 83.6 | 82.8 | 85.4 |
| 13 | *Cuora flavomarginata* | 74.6 | 85.0 | 91.8 | 87.7 | 87.7 | 88.0 | 87.3 | 83.3 |
| 14 | *Cuora galbinifrons* | 84.4 | 87.5 | 89.3 | 88.2 | 88.0 | 87.1 | 91.3 | 91.3 |
| 15 | *Geoclemys hamiltonii* | 89.3 | 93.7 | 95.8 | 93.5 | 94.2 | 93.5 | 95.0 | 95.3 |
| 16 | *Heosemys spinosa* | 83.3 | 89.4 | 85.1 | 93.9 | 93.5 | 84.6 | 89.0 | 84.3 |
| 17 | *Mauremys sinensis* | 75.2 | 89.8 | 83.1 | 89.7 | 82.7 | 77.0 | 80.9 | 81.6 |
| 18 | *Podocnemis unifilis* | 87.2 | 84.1 | 90.7 | 89.1 | 90.0 | 81.6 | 85.0 | 92.5 |
| 19 | *Aldabrachelys gigantea* | 92.5 | 91.6 | 95.0 | 95.8 | 94.3 | 92.9 | 92.8 | 94.3 |
| 20 | *Chelonoidis carbonaria* | 75.0 | 93.8 | 93.6 | 91.8 | 90.6 | 85.2 | 81.8 | 89.7 |
| 21 | *Chelonoidis chilensis* | 75.5 | 86.6 | 78.5 | 79.2 | 77.8 | 76.6 | 75.7 | 77.8 |
| 22 | *Chelonoidis denticulata* | 68.9 | 82.3 | 84.5 | 82.3 | 80.4 | 82.4 | 81.8 | 87.3 |
| 23 | *Chersina angulata* | 93.2 | 96.3 | 90.7 | 93.3 | 86.5 | 94.1 | 94.0 | 92.5 |
| 24 | *Geochelone elegans* | 82.3 | 79.3 | 86.3 | 85.0 | 84.9 | 84.5 | 89.8 | 84.7 |
| 25 | *Geochelone platynota* | 67.9 | 75.4 | 78.6 | 72.1 | 71.4 | 59.0 | 70.8 | 73.0 |
| 26 | *Geochelone sulcata* | 63.0 | 73.8 | 74.0 | 77.0 | 78.0 | 75.6 | 70.9 | 80.4 |
| 27 | *Gopherus berlandieri* | 90.3 | 94.6 | 89.2 | 92.1 | 94.5 | 91.1 | 94.2 | 94.9 |
| 28 | *Indotestudo elongata* | 77.1 | 83.9 | 84.9 | 72.5 | 73.8 | 75.8 | 73.5 | 63.8 |
| 29 | *Malacochersus tornieri* | 78.0 | 90.4 | 87.7 | 83.1 | 87.1 | 88.5 | 85.6 | 85.1 |
| 30 | *Manouria emys* | 76.4 | 82.9 | 80.6 | 85.4 | 85.3 | 76.9 | 84.2 | 91.5 |
| 31 | *Manouria impressa* | 75.3 | 84.4 | 86.5 | 86.0 | 82.0 | 76.3 | 87.2 | 81.7 |
| 32 | *Stigmochelys pardalis* | 95.9 | 97.6 | 97.6 | 97.6 | 97.3 | 97.4 | 97.6 | 97.4 |
| 33 | *Testudo horsfieldii* | 91.6 | 95.5 | 95.1 | 87.1 | 93.7 | 94.0 | 94.9 | 92.9 |
| 34 | *Testudo hermanni* | 89.1 | 95.2 | 94.6 | 96.7 | 91.9 | 93.8 | 88.9 | 95.1 |
| 35 | *Testudo marginata* | 77.9 | 94.5 | 88.1 | 88.2 | 85.0 | 83.9 | 86.2 | 82.8 |
| 36 | *Lissemys punctata* | 67.8 | 81.7 | 85.2 | 78.1 | 78.3 | 86.0 | 80.9 | 79.3 |

**Table S3.** Information about the species examined in this study.

| **Number** | **Family** | **Species** | **Native distribution** | **IUCN Red List** | **CITES Appendix** |
| --- | --- | --- | --- | --- | --- |
| 1 | Carettochelyidae | *Carettochelys insculpta* | Australia, Indonesia, Papua New Guinea | Endangered | Ⅱ |
| 2 | Chelidae | *Chelodina mccordi* | Indonesia, Timor-Leste | Critically Endangered | Ⅱ |
| 3 | Cheloiniidae | *Caretta caretta* | Subtropical and temperate regions of  the Mediterranean Sea and Pacific, Indian, and Atlantic Oceans | Vulnerable | Ⅰ |
| 4 | Cheloiniidae | *Chelonia mydas* | Tropical and, to a lesser extent, subtropical waters of  Atlantic, Indian, and Pacific Oceans | Endangered | Ⅰ |
| 5 | Cheloiniidae | *Eretmochelys imbricata* | Tropical and, to a lesser extent, subtropical waters of  Atlantic, Indian, and Pacific Oceans | Critically Endangered | Ⅰ |
| 6 | Cheloiniidae | *Macrochelys temminckii* | United States | Vulnerable | Ⅱ |
| 7 | Emydidae | *Clemmys guttata* | Canada, United States | Endangered | Ⅱ |
| 8 | Emydidae | *Graptemys ouachitensis* | United States | Least Concern | Ⅲ |
| 9 | Emydidae | *Malaclemys terrapin* | United States | Vulnerable | Ⅱ |
| 10 | Emydidae | *Terrapene carolina* | Canada, Mexico, United States | Vulnerable | Ⅱ |
| 11 | Emydidae | *Terrapene ornata* | Mexico, United States | Near Threatened | Ⅱ |
| 12 | Geoemydidae | *Cuora amboinensis* | Bangladesh, Brunei Darussalam, India, Indonesia, Lao People's Democratic Republic, Malaysia, Myanmar, Philippines, Singapore, Thailand, Viet Nam | Endangered | Ⅱ |
| 13 | Geoemydidae | *Cuora flavomarginata* | China, Japan, Taiwan, Province of China | Endangered | Ⅱ |
| 14 | Geoemydidae | *Cuora galbinifrons* | China, Lao People's Democratic Republic, Viet Nam | Critically Endangered | Ⅰ |
| 15 | Geoemydidae | *Geoclemys hamiltonii* | Bangladesh, India, Nepal, Pakistan | Endangered | Ⅰ |
| 16 | Geoemydidae | *Heosemys spinosa* | Brunei Darussalam, Indonesia, Malaysia, Myanmar, Philippines, Singapore, Thailand | Endangered | Ⅱ |
| 17 | Geoemydidae | *Mauremys sinensis* | China, Taiwan, Province of China, Viet Nam | Critically Endangered | Ⅲ |
| 18 | Podocnemididae | *Podocnemis unifilis* | Bolivia, Brazil, Colombia, Ecuador, French Guiana, Guyana, Peru, Suriname, Venezuela | Vulnerable | Ⅱ |
| 19 | Testudinidae | *Aldabrachelys gigantea* | Madagascar, Seychelles, Tanzania | Vulnerable | Ⅱ |
| 20 | Testudinidae | *Chelonoidis carbonaria* | Argentina, Bolivia, Brazil, Colombia, French Guiana, Guyana, Panama, Paraguay, Peru, Suriname, Venezuela | - | Ⅱ |
| 21 | Testudinidae | *Chelonoidis chilensis* | Argentina, Paraguay | Vulnerable | Ⅱ |
| 22 | Testudinidae | *Chelonoidis denticulata* | Argentina, Paraguay | Vulnerable | Ⅱ |
| 23 | Testudinidae | *Chersina angulata* | Namibia, South Africa | Least Concern | Ⅱ |
| 24 | Testudinidae | *Geochelone elegans* | India, Pakistan, Sri Lanka | Vulnerable | Ⅰ |
| 25 | Testudinidae | *Geochelone platynota* | Myanmar | Critically Endangered | Ⅰ |
| 26 | Testudinidae | *Geochelone sulcata* | Burkina Faso, Central African Republic, Chad, Eritrea, Ethiopia, Mali, Mauritania, Niger, Nigeria, Senegal, Sudan | Endangered | Ⅱ |
| 27 | Testudinidae | *Gopherus berlandieri* | Mexico, United States | Least Concern | Ⅱ |
| 28 | Testudinidae | *Indotestudo elongata* | Bangladesh, Bhutan, Cambodia, India, Lao People's Democratic Republic, Malaysia, Myanmar, Nepal, Thailand, Viet Nam | Critically Endangered | Ⅱ |
| 29 | Testudinidae | *Malacochersus tornieri* | Kenya, Tanzania, Zambia | Critically Endangered | Ⅰ |
| 30 | Testudinidae | *Manouria emys* | Bangladesh, India, Indonesia, Malaysia, Myanmar, Thailand | Critically Endangered | Ⅱ |
| 31 | Testudinidae | *Manouria impressa* | Cambodia, China, Lao People's Democratic Republic, Malaysia, Myanmar, Thailand, Viet Nam | Endangered | Ⅱ |
| 32 | Testudinidae | *Stigmochelys pardalis* | Angola, Botswana, Burundi, Congo, Djibouti, Eswatini, Ethiopia, Kenya, Malawi, Mozambique, Namibia, Rwanda, Somalia, South Africa, South Sudan, Tanzania, Uganda, Zambia, Zimbabwe | Least Concern | Ⅱ |
| 33 | Testudinidae | *Testudo horsfieldii* | Afghanistan, Armenia, Azerbaijan, China, Iran, Kazakhstan, Kyrgyzstan, Pakistan, Russian Federation, Tajikistan, Turkmenistan, Uzbekistan | Vulnerable | Ⅱ |
| 34 | Testudinidae | *Testudo hermanni* | Albania, Bosnia and Herzegovina, Bulgaria, Croatia, France, Greece, Italy, Montenegro, Romania, Serbia, Spain, Turkey | Near Threatened | Ⅱ |
| 35 | Testudinidae | *Testudo marginata* | Albania, Greece | Least Concern | Ⅱ |
| 36 | Trionychidae | *Lissemys punctata* | Bangladesh, India, Myanmar, Nepal, Pakistan | Vulnerable | Ⅱ |

**Table S4.** The number of augmentation rages and the final training set of the 36 turtles examined in this study.

| **Number** | **Family** | **Species** | **Augmentation**  **range** | **Final training set** |
| --- | --- | --- | --- | --- |
| 1 | Carettochelyidae | *Carettochelys insculpta* | 16 | 10,296 |
| 2 | Chelidae | *Chelodina mccordi* | 24 | 10,290 |
| 3 | Cheloiniidae | *Caretta caretta* | 23 | 10,246 |
| 4 | Cheloiniidae | *Chelonia mydas* | 20 | 10,332 |
| 5 | Cheloiniidae | *Eretmochelys imbricata* | 24 | 10,388 |
| 6 | Cheloiniidae | *Macrochelys temminckii* | 16 | 10,428 |
| 7 | Emydidae | *Clemmys guttata* | 17 | 10,500 |
| 8 | Emydidae | *Graptemys ouachitensis* | 23 | 10,246 |
| 9 | Emydidae | *Malaclemys terrapin* | 15 | 10,168 |
| 10 | Emydidae | *Terrapene carolina* | 14 | 10,034 |
| 11 | Emydidae | *Terrapene ornata* | 16 | 10,362 |
| 12 | Geoemydidae | *Cuora amboinensis* | 20 | 10,168 |
| 13 | Geoemydidae | *Cuora flavomarginata* | 23 | 10,340 |
| 14 | Geoemydidae | *Cuora galbinifrons* | 23 | 10,152 |
| 15 | Geoemydidae | *Geoclemys hamiltonii* | 14 | 10,388 |
| 16 | Geoemydidae | *Heosemys spinosa* | 23 | 10,058 |
| 17 | Geoemydidae | *Mauremys sinensis* | 24 | 10,034 |
| 18 | Podocnemididae | *Podocnemis unifilis* | 20 | 10,496 |
| 19 | Testudinidae | *Aldabrachelys gigantea* | 12 | 10,700 |
| 20 | Testudinidae | *Chelonoidis carbonaria* | 10 | 10,584 |
| 21 | Testudinidae | *Chelonoidis chilensis* | 22 | 10,080 |
| 22 | Testudinidae | *Chelonoidis denticulata* | 17 | 10,010 |
| 23 | Testudinidae | *Chersina angulata* | 21 | 10,406 |
| 24 | Testudinidae | *Geochelone elegans* | 19 | 10,530 |
| 25 | Testudinidae | *Geochelone platynota* | 20 | 10,250 |
| 26 | Testudinidae | *Geochelone sulcata* | 24 | 10,290 |
| 27 | Testudinidae | *Gopherus berlandieri* | 24 | 10,290 |
| 28 | Testudinidae | *Indotestudo elongata* | 23 | 10,058 |
| 29 | Testudinidae | *Malacochersus tornieri* | 17 | 10,290 |
| 30 | Testudinidae | *Manouria emys* | 24 | 10,290 |
| 31 | Testudinidae | *Manouria impressa* | 23 | 10,246 |
| 32 | Testudinidae | *Stigmochelys pardalis* | 13 | 10,368 |
| 33 | Testudinidae | *Testudo horsfieldii* | 17 | 10,290 |
| 34 | Testudinidae | *Testudo hermanni* | 24 | 10,290 |
| 35 | Testudinidae | *Testudo marginata* | 18 | 10,508 |
| 36 | Trionychidae | *Lissemys punctata* | 24 | 10,290 |

**Table S5**. The structure of VGGNet based on SSD architecture. Each "Conv" layer in the table corresponds to the composite function sequence Conv-ReLU.

| **Layers** | **Output Size (Width**$\boldsymbol{\times}$**Height**$\boldsymbol{\times}$**Channel)** | **Specification** |
| --- | --- | --- |
| Conv $\times$ 2 | 300$\times$300$\times$64 | 3$\times$3 Conv, stride 1, name : conv1_1 |
|  | 300$\times$300$\times$64 | 3$\times$3 Conv, stride 1, name : conv1_2 |
| Pooling | 150$\times$150$\times$64 | 2$\times$2 Max Pool, stride 2 |
| Conv $\times$ 2 | 150$\times$150$\times$128 | 3$\times$3 Conv, stride 1, name : conv2_1 |
|  | 150$\times$150$\times$128 | 3$\times$3 Conv, stride 1, name : conv2_2 |
| Pooling | 75$\times$75$\times$128 | 2$\times$2 Max Pool, stride 2 |
| Conv $\times$ 3 | 75$\times$75$\times$256 | 3$\times$3 Conv, stride 1, name : conv3_1 |
|  | 75$\times$75$\times$256 | 3$\times$3 Conv, stride 1, name : conv3_2 |
|  | 75$\times$75$\times$256 | 3$\times$3 Conv, stride 1, name : conv3_3 |
| Pooling | 38$\times$38$\times$256 | 2$\times$2 Max Pool, stride 2 |
| Conv $\times$ 3 | 38$\times$38$\times$512 | 3$\times$3 Conv, stride 1, name : conv4_1 |
|  | 38$\times$38$\times$512 | 3$\times$3 Conv, stride 1, name : conv4_2 |
|  | 38$\times$38$\times$512 | 3$\times$3 Conv, stride 1, name : conv4_3 |
| Pooling | 19$\times$19$\times$512 | 2$\times$2 Max Pool, stride 2 |
| Conv $\times$ 3 | 19$\times$19$\times$512 | 3$\times$3 Conv, stride 1, name : conv5_1 |
|  | 19$\times$19$\times$512 | 3$\times$3 Conv, stride 1, name : conv5_2 |
|  | 19$\times$19$\times$512 | 3$\times$3 Conv, stride 1, name : conv5_3 |
| Pooling | 19$\times$19$\times$512 | 3$\times$3 Max Pool, stride 1 |
| Weight parameters (unit: million) | | 25 |

**Table S6.** The structure of ResNet based on SSD architecture. Each "Conv" layer in the table corresponds to the composite function sequence BN-ReLU-Conv.

| **Layers** | **Output Size (Width**$\boldsymbol{\times}$**Height**$\boldsymbol{\times}$**Channel)** | **18-layer** | **34-layer** | **50-layer** |
| --- | --- | --- | --- | --- |
| Conv | 75$\times$75$\times$64 | 7$\times$7 Conv, stride 2  3$\times$3 Max Pool, stride 2 | | |
| Block (1) | 75$\times$75$\times$64 | $\left[ \begin{matrix} 3\times3 Conv \\ 3\times3 Conv \end{matrix} \right]\times2$ | $\left[ \begin{matrix} 3\times3 Conv \\ 3\times3 Conv \end{matrix} \right]\times3$ | $\left[ \begin{matrix} 1\times1 Conv \\ 3\times3 Conv \\ 1\times1 Conv \end{matrix} \right]\times3$ |
| Block (2) | 38$\times$38$\times$128 | $\left[ \begin{matrix} 3\times3 Conv \\ 3\times3 Conv \end{matrix} \right]\times2$ | $\left[ \begin{matrix} 3\times3 Conv \\ 3\times3 Conv \end{matrix} \right]\times4$ | $\left[ \begin{matrix} 1\times1 Conv \\ 3\times3 Conv \\ 1\times1 Conv \end{matrix} \right]\times4$ |
| Block (3) | 19$\times$19$\times$256 | $\left[ \begin{matrix} 3\times3 Conv \\ 3\times3 Conv \end{matrix} \right]\times2$ | $\left[ \begin{matrix} 3\times3 Conv \\ 3\times3 Conv \end{matrix} \right]\times6$ | $\left[ \begin{matrix} 1\times1 Conv \\ 3\times3 Conv \\ 1\times1 Conv \end{matrix} \right]\times6$ |
| Weight parameters (unit: million) | | 10 | 16 | 23 |

**Table S7**. The structure of DenseNet based on SSD architecture. Growth rate K = 32 was used for each dense block. Each "Conv" layer in the table corresponds to the composite function sequence BN-ReLU-Conv.

| **Layers** | **Output Size (Width**$\boldsymbol{\times}$**Height**$\boldsymbol{\times}$**Channel)** | **18-layer** | **30-layer** | **50-layer** | **121-layer** |
| --- | --- | --- | --- | --- | --- |
| Conv | 75$\times$75$\times$64 | 7$\times$7 Conv, stride 2  3$\times$3 max pooling, stride 2 | | | |
| Dense block (1) | 75$\times$75$\times$256 | $\left[ \begin{matrix} 1\times1 Conv \\ 3\times3 Conv \end{matrix} \right]\times6$ | $\left[ \begin{matrix} 1\times1 Conv \\ 3\times3 Conv \end{matrix} \right]\times6$ | $\left[ \begin{matrix} 1\times1 Conv \\ 3\times3 Conv \end{matrix} \right]\times8$ | $\left[ \begin{matrix} 1\times1 Conv \\ 3\times3 Conv \end{matrix} \right]\times6$ |
| Transition (1) | 75$\times$75$\times$256 | 1$\times$1 Conv | | | |
|  | 38$\times$38$\times$256 | 2$\times$2 average pooling, stride 2 | | | |
| Dense block (2) | 38$\times$38$\times$384 | $\left[ \begin{matrix} 1\times1 Conv \\ 3\times3 Conv \end{matrix} \right]\times4$ | $\left[ \begin{matrix} 1\times1 Conv \\ 3\times3 Conv \end{matrix} \right]\times8$ | $\left[ \begin{matrix} 1\times1 Conv \\ 3\times3 Conv \end{matrix} \right]\times10$ | $\left[ \begin{matrix} 1\times1 Conv \\ 3\times3 Conv \end{matrix} \right]\times12$ |
| Transition (2) | 38$\times$38$\times$384 | 1$\times$1 Conv | | | |
|  | 19$\times$19$\times$384 | 2$\times$2 average pooling, stride 2 | | | |
| Dense block (3) | 19$\times$19$\times$512 | $\left[ \begin{matrix} 1\times1 Conv \\ 3\times3 Conv \end{matrix} \right]\times4$ | $\left[ \begin{matrix} 1\times1 Conv \\ 3\times3 Conv \end{matrix} \right]\times8$ | $\left[ \begin{matrix} 1\times1 Conv \\ 3\times3 Conv \end{matrix} \right]\times13$ | $\left[ \begin{matrix} 1\times1 Conv \\ 3\times3 Conv \end{matrix} \right]\times24$ |
| Transition (3) without pooling | 19$\times$19$\times$512 | 1$\times$1 Conv | | | |
| Dense block (4) | 19$\times$19$\times$640 | $\left[ \begin{matrix} 1\times1 Conv \\ 3\times3 Conv \end{matrix} \right]\times4$ | $\left[ \begin{matrix} 1\times1 Conv \\ 3\times3 Conv \end{matrix} \right]\times8$ | $\left[ \begin{matrix} 1\times1 Conv \\ 3\times3 Conv \end{matrix} \right]\times19$ | $\left[ \begin{matrix} 1\times1 Conv \\ 3\times3 Conv \end{matrix} \right]\times16$ |
| Transition (4) without pooling | 19$\times$19$\times$640 | 1$\times$1 Conv | | | |
| Weight parameters (unit: million) | | 13 | 20 | 32 | 39 |
